# Current Practice in the Management of Pulmonary Nodules Detected on Computed Tomography Chest Scans

**DOI:** 10.1155/2019/9719067

**Published:** 2019-01-06

**Authors:** Clarus Leung, Tawimas Shaipanich

**Affiliations:** ^1^Department of Medicine, University of British Columbia, Vancouver, Canada; ^2^Division of Respiratory Medicine, University of British Columbia, Vancouver, Canada

## Abstract

Lung cancer is associated with high mortality. It can present as one or more pulmonary nodules identified on computed tomography (CT) chest scans. The National Lung Screening Trial has shown that the use of low-dose CT chest screening can reduce deaths due to lung cancer. High adherence to appropriate follow-up of positive results, including imaging or interventional approaches, is an important aspect of pulmonary nodule management. Our study is one of the first to evaluate the current practice in managing pulmonary nodules and to explore potential causes for nonadherence to follow-up. This is a retrospective analysis at St. Paul's Hospital, a tertiary healthcare center in Vancouver, British Columbia, Canada. We first identified CT chest scans between January 1 to June 30, 2014, that demonstrated one or more pulmonary nodules equal to or greater than 6 mm in diameter. We then looked for evidence of interventional (surgical resection or biopsy, or bronchoscopy for transbronchial biopsy and cytology) and radiological follow-up of the pulmonary nodule by searching on the province-wide CareConnect eHealth Viewer patient database. A total of 1614 CT reports were analyzed and 139 (8.6%) had a positive finding. Out of the 97 patients who received follow-up, 54.6% (*N* = 53) was referred for a repeat CT chest scan and 36.1% (*N* = 35) and 9.3% (*N* = 9) were referred for interventional biopsy and surgical resection, respectively. In our study, 30.2% (*N* = 42) of the patients with pulmonary nodules were nonadherent to follow-up. Despite the radiologist's recommendation for follow-up within a certain time interval, only 36% had repeat imaging in a timely manner. Our findings reflect the current practice in the management of pulmonary nodules and suggest that there is a need for improvement at our academic center. Adherence to follow-up is important for the potentially near-future implementation of lung cancer screening.

## 1. Introduction

Pulmonary nodules are heterogeneous and nonspecific, representing a spectrum of benign and malignant etiologies. Currently, radiologists utilize the Fleischner Society 2017 guidelines in risk stratification of detected nodules and recommendation of appropriate imaging surveillance interval. Risk stratification is based on certain radiological features including most notably, nodule size, as well as marginal spiculation, nodule location, and growth rate, and presence of concurrent emphysema or pulmonary fibrosis. Patient risk factors are also important, such as age, family history, smoking history, and exposure to asbestos, uranium, and radon [1, 2]. Overall, the estimated risk of cancer in nodules smaller than 6 mm is less than 1% [1]. The probability of malignancy in lung nodules detected on baseline screening low-dose CT scans ranges from 3.7 to 5.5% [3].

Lung cancer is the second most common malignancy in Canada for both men and women and is the leading cause of cancer-related deaths worldwide [4, 5]. Despite advancement in diagnostic and therapeutic approaches in the last few decades, survival from lung cancer has not significantly improved. One of the reasons may be that lung cancer is often identified at an advanced stage associated with metastases. Due to the aggressive nature of this disease, efforts have been directed toward early detection through screening. In 2011, the National Lung Screening Trial (NLST) was conducted to determine the impact of low-dose CT versus single-view posteroanterior chest radiography on lung cancer mortality in high-risk group aged 55–74 with at least 30 pack-years smoking history, or if former smokers, having quit within the previous 15 years. This landmark study demonstrated that the relative reduction of mortality from lung cancer with low-dose CT screening in this population was 20% (95% CI, 6.8 to 26.7; *P*=0.004) and the all-cause mortality reduction was 6.7% [6]. The adherence rate in the NLST was more than 90%, which illustrates the importance of having a well-established setting with appropriate resources in the management of detected pulmonary nodules, including follow-up imaging and referral for intervention.

In 2016, the Canadian Task Force updated its recommendation to include annual low-dose computed tomography up to three consecutive times for the high-risk group as indicated in the NLST [7]. However, this guideline strongly emphasizes that lung cancer screening should only be conducted in a healthcare center with established expertise in early diagnosis and treatment of lung cancer.

The University Health Network in Toronto has conducted a lung cancer screening trial with optimistic preliminary results, including a high proportion of early stage diagnoses and a high rate of resection in those diagnosed with lung cancer [8]. The British Columbia Cancer Agency is currently recruiting patients for the ongoing BC Lung Screen Trial [9]. The Pan-Canadian Lung Screening study across 8 Canadian centers prospectively recruited individuals for lung cancer screening on the basis of a predictive risk model and demonstrated a high cumulative incidence of lung cancer (4.0%) compared to the NLST and a significantly larger proportion (77%) of early stage (I or II) lung cancer [10].

According to cost analyses conducted from the Canadian public payer's perspective using the Pan-Canadian Early Detection of Lung Cancer (PanCan) study data, the average cost to screen individuals with a high risk of developing lung cancer using low-dose CT and the average initial cost of curative intent treatment were lower than the average per-person cost of treating advanced staged lung cancer [11]. The high volume of low-dose CT scans used to screen the general population based on age and smoking history criteria as in the NLST could have substantial budgetary impacts. Therefore, Cressman et al. retrospectively identified high-risk participants in the NLST using the risk prediction tool developed from the Prostate, Lung, Colorectal and Ovarian Cancer Screening Trial (PLCO) and compared them with the low-risk subgroup and the participants in the PanCan study. The research group found that the drivers of program cost-effectiveness were non-lung cancer outcomes such as mortality reduction and quality of life. High-risk screening would cost $20,724 (in 2015 Canadian dollars) per quality-adjusted life year gained. Moreover, lung cancer screening may be a cost-saving intervention as the costs of noncurative drugs and therapies increase [12].

As lung cancer screening is one step closer to implementation, we were interested in analyzing the current practice in managing positive findings of pulmonary nodules at our center. In particular, our study looked at the adherence rate to follow-up imaging or investigations which can be influenced by multiple individual and system factors.

## 2. Materials and Methods

### 2.1. Study Design

This study is a retrospective analysis of CT chest scans completed at St. Paul's Hospital in Vancouver, British Columbia, Canada between January 1 and June 30, 2014. Our hospital is a tertiary referral center that serves a heterogeneous, multiracial, inner city population in downtown Vancouver. Institutional ethics committee approval was obtained (H17-01105, University of British Columbia).

### 2.2. Imaging Acquisition

The CT chest images were available in the InteleViewer PACS system, using the search filter criteria “chest” and specified “CT” imaging modality. This search yielded various CT chest imaging protocols, including high resolution CT, pulmonary embolism, lung nodule, and thoracic aorta scans with and without the use of intravenous contrast. All CT chest studies were obtained with helical technique by using GE Discovery HD 750, GE Lightspeed VCTXT, and GE Revolution (GE Healthcare, Milwaukee, WI, USA) CT scanner. CT images were obtained with the following parameters: tube voltage, 100 kVp (for BMI < 30) and 120 kVp (for BMI > 30); tube rotation, 0.5–1.0 sec; tube current, 20 mA; reconstruction thickness, 1.25–2.5 mm.

### 2.3. Identification of Pulmonary Nodules

Our inclusion criterion was one or more nodules within the lung parenchyma or pleura with the largest dimension equal to or greater than 6 mm in a CT chest study without previous radiological evidence of pulmonary nodules, lung cancer, or metastasis. If there were multiple nodules identified, then the largest dimension taken from the largest nodule was recorded. The size threshold of 6 mm was chosen to reflect the Fleischner Society 2017 guidelines which recommended imaging follow-up in all solid and ground-glass pulmonary nodules without benign features above this size limit [1]. We included nodules of all shape (round or nonround), location (subpleural, perifissural, and parenchymal), margin (smooth, lobulated, and spiculated), and density except benign calcification (solid, part solid, or ground-glass opacity). Patients over the age of 18 were included.

### 2.4. Pulmonary Nodule Follow-Up

After identifying patients with one or more pulmonary nodules that meet our study criteria, we searched for evidence of follow-up at a later time which is defined as repeat CT chest imaging indicated for pulmonary nodule follow-up, surgical resection or biopsy of the lung nodule or suspected lung metastatic disease, or bronchoscopy for cytology or transbronchial tissue biopsy. The search for follow-up was completed using the CareConnect eHealth Viewer system which is a provincial patient database for imaging, laboratory, or pathology results in British Columbia, Canada. We searched for CT chest imaging, pathology, or cytology reports that were completed after the initial positive finding. The date on which the pathology or cytology sample was collected was recorded as the date of interventional follow-up. The occurrence of surgical resection such as lobectomy was noted from the pathology records. The outcomes of the study were the rate of pulmonary nodule follow-up and the time to completion of radiological or interventional follow-up.

### 2.5. Statistical Analyses

The two-sample *t*-test was used to analyse the difference between the two groups (follow-up vs. lost to follow-up) and nodule size. The chi-squared test was used to analyse the difference in patient age and sex. Fisher's exact test was used to examine the differences in the city of residence and primary language between the two groups. A *P* value < 0.05 was considered to indicate statistical significance. Data were analyzed using SAS Software.

## 3. Results

Baseline patient characteristics are described in Table 1. There was no significant difference in mean age or distribution in sex between the patients adherent vs. nonadherent to follow-up. The majority of the patient population from both groups resided in the city of Vancouver. Some patients in this study lived in other Canadian provinces and territories such as Alberta and Yukon. Most of the patients in our study population communicated in English as their primary language. Overall, there was no significant difference between the two groups in city of residence or primary language. The size of the pulmonary nodules was significantly larger (19.82 mm vs. 12.94 mm, *P*=0.01) in the follow-up group.

**Figure 1:**
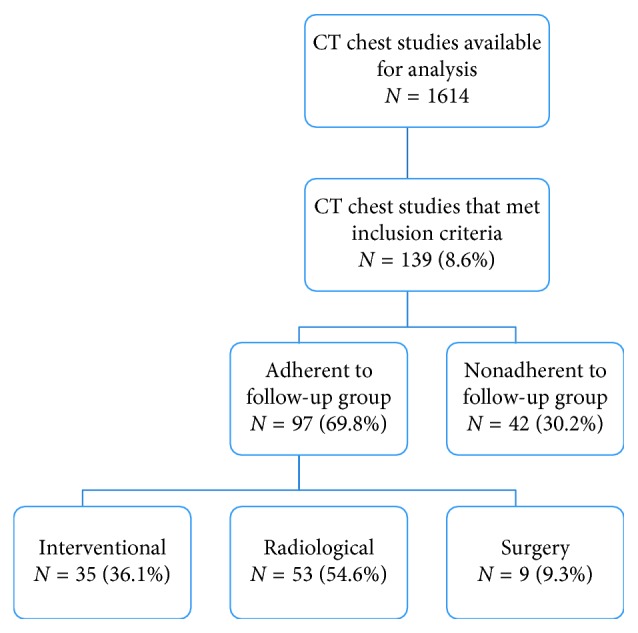
Study profile and rate of pulmonary nodule follow-up. Interventional follow-up refers to surgical and/or transbronchial biopsy or cytology from bronchoscopy. Radiological follow-up refers to a repeat CT chest imaging indicated for nodule assessment after an interval time. Several patients underwent surgical resection of the pulmonary nodule(s).

A total of 1614 CT chest reports completed between January 1 to June 30, 2014, were analyzed, and 139 (8.6%) met inclusion criteria. Out of the 97 patients who received follow-up, 36.1% (*N* = 35) were referred for interventional biopsy including surgical and transbronchial biopsies. In addition, 9.3% (*N* = 9) and 54.6% (*N* = 53) were referred for surgical resection and repeat CT chest scan, respectively (Figure 1). In our study, 30.2% (*N* = 42) of the patients with pulmonary nodules were nonadherent to any form of follow-up.

In the interventional follow-up group, the majority (88.6%, *N* = 31) underwent surgical biopsy and the rest had bronchoscopy. Analysis of the pathology results revealed that 75% (*N* = 3) was diagnosed with a primary lung malignancy in the bronchoscopy subgroup. Among the patients who underwent surgical biopsy or resection, 63% (*N* = 25) was diagnosed with a primary lung malignancy in the surgical subgroup.

**Figure 2:**
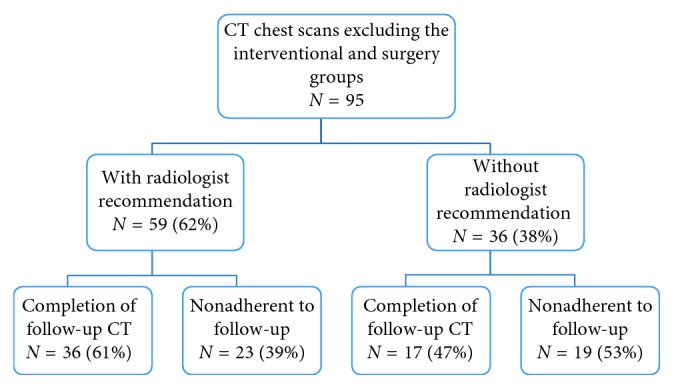
Analysis of follow-up in CT chest reports with vs. without an explicit imaging follow-up time interval recommendation by the interpreting radiologist. The rates of imaging follow-up were compared between the two groups, and this difference was not significant (*P*=0.19).

In patients who did not undergo surgery or bronchoscopy, the CT chest report was analyzed for an explicit comment regarding imaging follow-up recommendation within a time interval by the interpreting radiologist. We found that an explicit recommendation was provided in only 62% (*N* = 59) of the cases. In this group, only 36 of 59 (61%) had a repeat CT chest scan (Figure 2). When the radiologist did not make a specific recommendation (38%), the follow-up rate was 47%, *P*=0.19, 95% CI (32%, 63%) (Figure 2).

**Figure 3:**
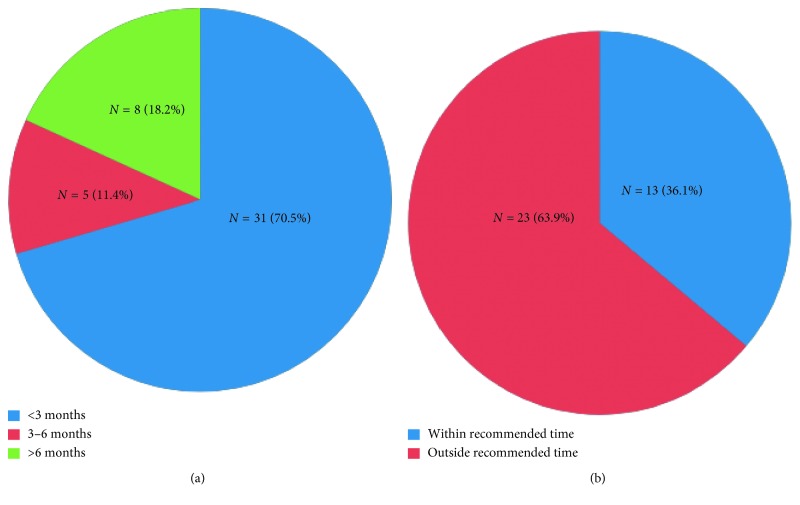
Time to receiving interventional, surgical, and radiological follow-up. In the combined surgical and interventional group, a majority (70.5%) of the patients underwent surgical resection or biopsy or transbronchial biopsy or cytology within 3 months (a). In the radiological follow-up group, 36.1% of the patients had a follow-up CT chest within the interpreting radiologist's recommended time interval (b).

In terms of the duration of time to receiving interventional or surgical follow-up, the majority (70.5%) underwent the procedure within 3 months, whereas 11.4% and 18.2% underwent the procedure between 3 and 6 months and after 6 months, respectively (Figure 3(a)). Overall, the mean time to completion of lung resection or surgical and interventional follow-up was found to be 173 days, 95% CI (69, 276 days). In the radiological follow-up group, the mean time to completion of repeat CT scan is 239 days, 95% CI (175, 303 days). Despite the radiologist's explicit recommendation for follow-up within a certain interval, only 13 of 36 (22%) underwent repeat imaging within the recommended time frame (Figure 3(b)).

## 4. Discussion

Our study is one of the first to analyse the current practice in the management of pulmonary nodules identified on CT chest studies at a Canadian tertiary healthcare center. In the NLST where screening was conducted in a high-risk population, the rate of positive finding, defined as a pulmonary nodule above the size threshold of 4 mm, was 24.2% [6]. This was much higher than our nodule prevalence of 8.6% due to the difference in size threshold and patient population. Other studies have estimated the prevalence of lung nodules in North America to be around 23% [2]. There are various routes that lead to detection of pulmonary nodules including lung cancer screening, clinical presentation of respiratory symptoms, and incidental findings in studies done for other purposes; however, this is not an important factor in management [2]. According to the Fleischner Society 2017 guidelines, all nodules without benign features and greater than 6 mm require follow-up imaging in both low-risk and high-risk populations [1]. We also found that basic demographic factors were similar in both groups (adherent vs. non-adherent to follow-up); however, pulmonary nodule size was significantly larger in the former group. This finding seems intuitive as larger nodules are more concerning for malignancy and therefore, are more likely to prompt further investigation.

We found that the overall follow-up rate is low, with only one-third of the patients receiving some form of interventional, surgical, or radiological follow-up for their pulmonary nodule. Despite an explicit recommendation from the interpreting radiologist for interval repeat imaging, the rate of follow-up was 61%. We did not find a significant difference in follow-up between the groups with and without radiologist's recommendation. This result was surprising as one would expect that a clear recommendation in the imaging report, which would be communicated back to the primary care physician, is one of the most important steps in the pulmonary nodule management. A possible reason for our finding may be that our study was inadequately powered to illustrate the difference. More importantly, this suggests that there are other system factors contributing to the overall low adherence rate. Another study has also demonstrated similarly low rates of adherence at 29% despite radiologist's recommendation [13]. Interestingly, having the radiologist's recommendation written in the impression summary rather than in the body of the report seems to be helpful in increasing follow-up rates [13].

In an ideal pulmonary nodule management pathway, the positive finding is clearly flagged and risk-stratified by the interpreting radiologist to determine the appropriate interval for follow-up imaging. The report is effectively communicated to the ordering physician who often may be an emergency physician or inpatient hospitalist and hence requiring an additional step in handing over to the patient's primary care physician responsible for organizing follow-up. This pathway is also dependent on physician adherence, patient compliance, and resource availability of healthcare centers, imaging technicians, and CT scanners. Barriers in this pathway can delay repeat imaging, as we have demonstrated in our study that only 36% of the patients received follow-up imaging within the recommended time frame.

To date, there is no clear recommendation regarding the optimal time to undergoing interventional investigation, such as surgical or transbronchial biopsy. The majority of our patients had interventional and surgical diagnostics within 3 months. However, it is uncertain whether undergoing biopsy in less than 3 months as compared to greater than 6 months has a significant impact on lung cancer outcome and mortality.

There were several limitations in our study. Firstly, we only analyzed the current practice in management at one tertiary hospital (St Paul's Hospital), and therefore the findings may not be generalizable to other facilities. Also, a reasonable proportion of patients at this hospital resided in Vancouver Downtown Eastside which is an impoverished area associated with low socioeconomic status [14]. This factor can affect the adherence rate to follow-up. In addition, we utilized the CareConnect eHealth Viewer database in our retrospective analysis. This database included most health authorities in British Columbia but excludes private facilities, rural areas, and other Canadian provinces. This may have led to underestimation of the follow-up rates if the patients underwent surveillance or diagnostic procedures at these sites. In our study, we had only assessed the first follow-up CT study and did not analyse the rates of subsequent surveillance studies. Finally, our study may have lacked sufficient power to illustrate significant differences between the groups, and thus it would be interesting to expand the scope of the study in attempt to identify other factors contributing to low rates of follow-up. This is potentially an area of future research.

Previous studies have shown that patient perspectives and shared decision making are important factors for successful cancer screening [15, 16]. As the majority of the CT lung scans analyzed in this study was ordered for reasons other than primary lung screening, patients may not have perceived the importance of follow-up of the incidentally discovered pulmonary nodule. For instance, some CT chest scans were completed to rule out pulmonary embolism in context of a symptomatic patient. The incidental finding of a nodule may be easily overlooked. These symptomatic patients would also be excluded from a screening test. Therefore, this limits the generalizability of our data to lung cancer screening.

Physician recommendation is a critical predictor of patient screening behaviours [15]. A qualitative study found that most patients are willing to undergo low-dose CT screening but many are unaware of why they are being screened and could not communicate the risks or benefits of the test [16]. Altogether, physicians' knowledge of guideline recommendations and thorough discussion for shared decision making are important factors for cancer screening. These aspects, although not accounted for in our study, are crucial aspects that require further research.

Lung cancer screening and management is a complex process integrating primary care physicians, respirologists, thoracic surgeons, medical and radiation oncologists, and palliative care specialists. Known challenges in lung cancer screening include radiation risk, high rate of false positives, patient psychological impact, difficulties in determining optimal eligibility criteria for screening, equitable access to screening, and cost [17]. In order for effective screening to occur at a population level, there needs to be a well-established referral system in place to manage suspicious pulmonary nodules.

Our study demonstrated a low adherence rate to follow-up imaging or interventional investigation for pulmonary nodules greater than 6 mm identified on CT chest scans at our healthcare facility. These findings reflect the current reality of current pulmonary nodule management and also strongly suggest a need for improvement. A structured referral system may be helpful in appropriately triaging and monitoring pulmonary nodules that can potentially develop into advanced lung cancer.

## 5. Conclusions

There is a need for improvement in the current practice of pulmonary nodule management at our tertiary healthcare center.

## Figures and Tables

**Table 1:** Baseline patient characteristics of study population (*n* = 139) in the adherent to follow-up vs. nonadherent to the follow-up group.

	Patients adherent to follow-up (*N* = 97)	Patients nonadherent to follow-up (*N* = 42)	*P* value
Mean age (yrs)	65.89	62.57	0.23
Sex (%)			0.98
Female	40.2% (*N* = 39)	40.5% (*N* = 17)	
Male	59.8% (*N* = 58)	59.5% (*N* = 25)	
City of residence			0.18
Within Vancouver	58.8% (*N* = 57)	71.4% (*N* = 30)	
Outside of Vancouver	41.2% (*N* = 40)	28.6% (*N* = 12)	
Primary language			0.46
English	85.6% (*N* = 83)	78.6% (*N* = 33)	
Other languages	14.4% (*N* = 14)	18.1% (*N* = 8)	
Unknown	0	2.4% (*N* = 1)	
Size of nodules (mm)	19.82	12.94	0.01

## Data Availability

The raw data used to support the findings of this study available from the corresponding author upon request.
